# Genetic Differences between Avian and Human Isolates of *Candida dubliniensis*

**DOI:** 10.3201/eid1509.081660

**Published:** 2009-09

**Authors:** Brenda A. McManus, Derek J. Sullivan, Gary P. Moran, Christophe d’Enfert, Marie-Elisabeth Bougnoux, Miles A. Nunn, David C. Coleman

**Affiliations:** Dublin Dental School and Hospital, Dublin, Ireland (B.A. McManus, D.J. Sullivan, G.P. Moran, D.C. Coleman); Trinity College Dublin, Dublin (B.A. McManus, D.J. Sullivan, G.P. Moran, D.C. Coleman); Institut Pasteur, Paris, France (C. d’Enfert, M.-E. Bougnoux); National Environmental Research Council Centre for Ecology and Hydrology, Oxford, UK (M.A. Nunn)

**Keywords:** Candida dubliniensis, multilocus sequencing typing, MLST, genetics, fungi, epidemiology, seabirds, dispatch

## Abstract

When *Candida dubliniensis* isolates obtained from seabird excrement and from humans in Ireland were compared by using multilocs sequence typing, 13 of 14 avian isolates were genetically distinct from human isolates. The remaining avian isolate was indistinguishable from a human isolate, suggesting that transmission may occur between humans and birds.

*Candida dubliniensis* is an opportunistic yeast species phenotypically and genetically closely related to *C*. *albicans*, the most common cause of *Candida* infection. However, *C*. *dubliniensis* is less pathogenic and is most commonly associated with superficial infection in immunocompromised persons. Although *C*. *albicans* has frequently been isolated from avian and animal sources (*1*–*4*), the recent study by Nunn et al. identified *C*. *dubliniensis* from a nonhuman source (*5*). These isolates were obtained from the surface of *Ixodes uriae* ticks that lived in cracks filled with seabird excrement at 2 locations at a seabird breeding colony on Great Saltee Island off the southeastern coast of Ireland.

Multilocus sequence typing (MLST) is an informative tool for investigating the population structure and epidemiology of many bacterial and fungal species (*6*). We have used MLST to show that *C*. *dubliniensis* has less genetic diversity than *C*. *albicans* and that *C*. *dubliniensis* isolates comprise 3 distinct clades (C1, C2, and C3), which correspond to described internally transcribed spacer (ITS) region genotypes 1–4 (*7*). Two other research groups recently used MLST to show genetic differences between *C*. *albicans* isolates from humans and animals (*3*,*4*). The purpose of our study was to use MLST, the presence or absence of a previously identified polymorphism in the *CDR1* gene (*8*), and mating type analysis to determine genetic relatedness between avian-associated and human *C*. *dubliniensis* isolates and whether avian-associated isolates are a source of human opportunistic infections.

## The Study

To obtain avian-associated *C*. *dubliniensis* isolates from a novel geographic site, fresh seabird excrement was sampled from the campus of Trinity College Dublin, ≈150 km north of Great Saltee Island by using nitrogen-gassed VI-PAK sterile swabs (Sarstedt-Drinagh, Wexford, Ireland). Samples were plated within 2 h of collection on CHROMagar *Candida* medium (CHROMagar, Paris, France), incubated at 30°C for 48 h, and identified as described (*7*,*9*–*12*). Three new *C*. *dubliniensis* isolates were obtained from 134 fecal samples. Like isolates from Great Saltee Island (*5*), these 3 isolates obtained directly from freshly deposited herring gull (*Larus argentatus*) excrement were ITS genotype 1 (*13*). Because the isolates originally described by Nunn et al. (*5*) were obtained from the surface of ticks living in avian excrement, avian-associated isolates refers to avian excrement–associated isolates. The avian-associated isolates were compared with 31 human *C*. *dubliniensis* strains belonging to MLST clade C1 as previously reported (*7*), and 5 additional *C*. *dubliniensis* clade C1 human isolates from Ireland (Table).

**Table:** Newly investigated avian-associated and human isolates of *Candida dubliniensis*, Ireland*

Isolate	Source	Year of isolation	Location	DST†	Mating type	TAG	Reference
SL411	*Ixodes uriae* ticks	2007	GSI	27	aa	+	(*5*)
SL422	*I. uriae*	2007	GSI	27	aa	+	(*5*)
SL370	*I. uriae*	2007	GSI	27	aa	+	(*5*)
SL410	*I. uriae*	2007	GSI	29	aa	+	(*5*)
SL375-I	*I. uriae*	2007	GSI	31	aa	+	(*5*)
SL375-II	*I. uriae*	2007	GSI	31	aa	+	(*5*)
SL397	*I. uriae*	2007	GSI	31	aa	+	(*5*)
SL414	*I. uriae*	2007	GSI	31	aa	+	(*5*)
SL495	*I. uriae*	2007	GSI	33	aa	+	(*5*)
SL509	*I. uriae*	2007	GSI	30	aa	+	(*5*)
SL522	*I. uriae*	2007	GSI	31	aa	+	(*5*)
AV5	*Larus argentatus‡*	2008	TCD	29	aa	+	This study
AV6	*L. argentatus*	2008	TCD	27	aa	+	This study
AV7	*L. argentatus*	2008	TCD	2	aα	+	This study
CD06032	Human, oral	2006	Ireland	36	αα	–	This study
CD06027	Human, oral	2006	Ireland	1	aα	+	This study
CD0512	Human, oral	2005	Ireland	37	aα	–	This study
CD524	Human, oral	1997	Ireland	35	aα	–	(*13*)
CD505	Human, oral	1989	Ireland	28	αα	+	(*13*)

*All 19 isolates were internal transcribed spacer genotype 1 and belonged to multilocus sequence typing clade C1. DST, diploid sequence type; TAG, TAG polymorphism; GSI, Great Saltee Island off the southeastern coast of Ireland; TCD, Trinity College Dublin. †Assigned to each isolate according the recommended multilocus sequence typing scheme for *C. dubliniensis* (scheme D) (*7*). All DSTs, except for DST1 and DST 2, are new. ‡Herring gull.

Isolates were assigned a diploid sequence type (DST) on the basis of genotype numbers for the 8 loci in the recommended *C*. *dubliniensis* MLST typing scheme (*7*) (Table). Six new DSTs were identified in 13 of 14 avian-associated isolates because of the identification of 2 new *exZWF1b* alleles that were found exclusively in avian- associated isolates. DST2 was the only previously identified DST (isolate AV7, Table). DST 31 was the most frequently (5/14 isolates) found DST in avian-associated *C*. *dubliniensis* isolates, all 5 of which were from Great Saltee Island (*5*). Four isolates belonged to DST 27, three from Great Saltee Island and 1 from Dublin (Table).

Polymorphic sites (n = 36) from the 8 MLST loci (*7*) of all 50 clade C1 human and avian-associated *C*. *dubliniensis* isolates were concatenated and used to construct a neighbor-joining tree (MEGA software program version 3.1 [*14*]), which included all known clade C1 DSTs identified. Thirteen of 14 avian-associated *C. dubliniensis* isolates, 11 from Great Saltee Island (*5*) and 2 from Dublin, formed a distinct subgroup within clade C1 (Figure, panel A). This same subgroup was also identified in trees generated by using the unweighted pair group method with arithmetic mean, maximum parsimony, and maximum likelihood, and based on related sequence types (BURST) analysis. To test for genetic separation between human and avian-associated isolates obtained from the same country, a neighbor-joining tree was constructed by using 13 avian-associated and human clade C1 isolates from Ireland, each of which represented unique DSTs. The tree showed the robustness of the avian-associated subgroup within a population of human isolates from the same country, and the distribution of avian-associated and human isolates differed significantly (p = 0.025, by Fisher exact test) (www.exactoid.com/fisher/index.php) (Figure, panel B).

**Figure:**
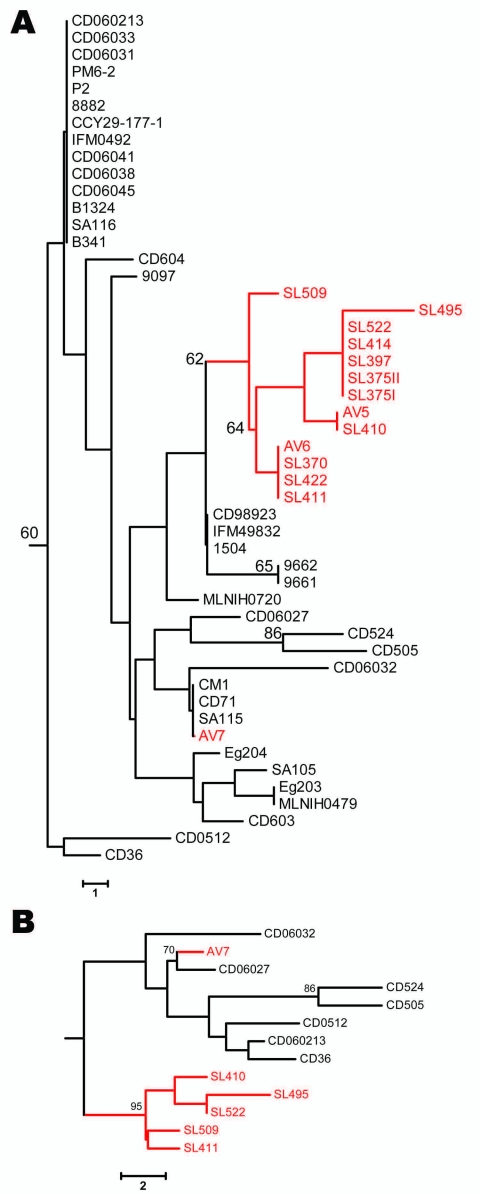
Neighbor-joining trees based on the polymorphic sites in *Candida dubliniensis* multilocus sequence typing (MLST) sequences. Bootstrap values >60% are indicated at cluster nodes. Avian-associated isolates are indicated in red. Numbers of polymorphic sites in isolates are indicated by scale bars. A) Isolates of MLST clade C1 defined by McManus et al. (*7*) showing location of avian-associated isolates in relation to human isolates in the same clade; human isolates were originally obtained in many countries. B) Neighbor-joining tree based on polymorphic sites in MLST sequences for each of 13 internal transcribed spacer genotype 1 *C. dubliniensis* isolates, 7 of which were obtained from humans in Ireland and 6 from seabird excrement in Ireland. Isolates that had identical diploid sequence types (DSTs) were not included in the tree so that only 1 of each DST is included. Tree displays the robustness of the avian-associated subgroup of isolates within a population of similar human-associated isolates from the same region. The rate of heterozygosity among human and avian-associated clade C1 isolates was 1.6 and 1 heterozygous site per DST, respectively, from 36 polymorphic sites, which indicated that avian-associated isolates were more clonal.

The prevalence of a common point mutation, previously identified in the *CDR1* gene of some ITS genotype 1 isolates, was determined for avian and human *C*. *dubliniensis* isolates as described previously (*8*). All 14 avian-associated isolates had the TAG polymorphism (Table) compared with 19 (53%) of 36 human clade C1 isolates. The mating types of the isolates were determined by multiplex PCR amplification by using 2 pairs of mating type locus (MTL)–specific primers. A 535-bp amplimer was generated with primers MTLa1-F (5′-TGAAAATGAAGACAATGCGA-3′) and MTLa1-R (5′-CATCTTTTTCTGCTATCAATTC-3′) in the presence of MTL type a DNA, and a 615-bp product resulted from primers MTLα1-F (5′-ATGAATTCACATCTGGAGGC-3′) and MTLα1-R (5′-CTGTTAATAGCAAAGCAGCC-3′) in the presence of MTL type α DNA. Amplification reactions contained 10 pmol of each of the forward and reverse primers, 2.5 mmol/L MgCl_2_, 10 mmol/L Tris-HCl, pH 9.0 at 25°C, 10 mmol/L KCl, 0.1% (vol/vol) Triton X-100, 1.25 U GoTaq polymerase (Promega, Madison, WI, USA), and 25 μL of template DNA in a total volume of 50 μL. Cycling conditions were at 94°C for 10 min; 30 cycles at 94°C for 1 min, 55°C for 2 min, and 72°C for 3 min; and a final step at 72°C for 10 min.

Of the 14 avian excrement–associated isolates, 13 were MTLa homozygous (a/a), (Table). Only 4 (11.1%) of 36 human clade C1 isolates were homozygous for MTLa; 28 (77.7%) of 36 were heterozygous for MTL (a/α). A previous study also reported that 17 (20.7%) of 82 human *C*. *dubliniensis* isolates were homozygous for MTLa (*15*). The gull isolate AV7 was indistinguishable from human isolates CD71, SA115, and CM1 by MLST and has the same mating type (a/α). We propose that AV7 may be a human isolate that colonized a gull scavenging on the Trinity College Dublin campus. The TAG polymorphism and mating type data from the avian-associated isolates suggest a highly clonal population.

## Conclusions

The avian-associated *C*. *dubliniensis* isolates investigated belong to MLST clade C1, which includes most human isolates. However, most (13/14) of the avian-associated isolates form a distinct subgroup within this clade, which suggests that despite the low level of variation within *C*. *dubliniensis*, a distinct avian subpopulation may be present. This suggestion is supported by the observation that 2/3 isolates (AV5 and AV6) obtained in Dublin belonged to the same subpopulation (defined by MLST, *CDR1*, and MTL loci) as isolates obtained from Great Saltee Island, which is 150 km from Dublin. Similar data suggesting genetic separation and differential clade distribution between human and animal populations of *C*. *albicans* have been reported (*3*,*4*). The presence of the avian-associated subgroup within the most predominant clade (C1), which had previously only been identified in human isolates, and the close genetic relatedness between isolates, in particular gull isolate AV7, suggests that transmission between the 2 hosts can occur. However, in this instance the most likely direction of transfer is from human to bird.
